# Using confirmatory factor analysis to identify symptom clusters in individuals with acquired disabilities

**DOI:** 10.1007/s11136-026-04220-0

**Published:** 2026-04-01

**Authors:** David S. Tulsky, Aaron J. Boulton, Pamela A. Kisala, Callie E. Tyner, Ryan Pohlig, Nancy D. Chiaravalloti, Jerry Slotkin, Heather B. Taylor, Mark Sherer

**Affiliations:** 1 Center for Health Assessment Research and Translation, University of Delaware, Newark, DE USA; 2 Departments of Physical Therapy and Psychological & Brain Sciences, University of Delaware, Newark, DE USA; 3 Department of Epidemiology, University of Delaware, Newark, DE USA; 4 Center for Neuropsychology and Neuroscience, Kessler Foundation, East Hanover, USA; 5 Department of Physical Medicine and Rehabilitation, Rutgers University, New Brunswick, USA; 6 University of Texas Health Science Center, Houston, TX USA; 7 Brain Injury Research Center, TIRR Memorial Hermann, Houston, TX USA; 8 Casa Colina Research Institute, Pomona, CA USA

**Keywords:** Symptom cluster, Cluster analysis, Factor analysis, Spinal cord injuries, Brain injuries, traumatic, Stroke, Amputation, Surgical, Patient reported outcome measures

## Abstract

**Purpose:**

Acquired disabilities, such as spinal cord injury, traumatic brain injury, limb loss (or other major limb injury/illness), or stroke, are typically accompanied by an array of physical and emotional symptoms and social challenges that interfere with recovery and affect quality of life (QOL). Despite advances in personalized medicine, interrelationships between symptoms remain unclear, and clinicians often treat individual symptoms in isolation. The objective of this study was to identify clusters of symptoms that occur across sudden-onset disability populations, with a long-term goal of identifying targets for clinical intervention.

**Methods:**

Participants were 755 adults who sustained a spinal cord injury, traumatic brain injury, stroke, or sudden-onset limb-threatening injury/illness. Participants completed a battery of physical, emotional, and social patient reported outcomes measures and performance-based cognitive tests at baseline and after 14–18 months. Confirmatory factor analysis (CFA) of 26 indicator variables was used to reduce dimensions and identify clusters of symptoms.

**Results:**

CFA results provide support for 10 factors: Cognition, Economic QOL, Negative Affect, Positive Affect, Psychological Adjustment, Physical Function, Independence, Sleep Impairment/Fatigue, Pain, and Social Health. Global model fit was acceptable: χ^2^ (255) = 875.0, RMSEA = 0.059, CFI = 0.948. The three emotional health clusters were highly correlated. The 10 factors demonstrated stability over time.

**Conclusion:**

These results can inform the development of composite scores and/or symptom indices to identify and compare broad areas of functioning and provide a method to detect specific areas of clinical concern. This improved understanding of symptom interrelationships may facilitate individualized care based on specific patient characteristics and, ultimately, lead to improved clinical outcomes for individuals with acquired disabilities.

**Supplementary Information:**

The online version contains supplementary material available at 10.1007/s11136-026-04220-0.

The twenty-first century has seen drastic improvements in the way patients’ experiences of their health and symptoms are measured and considered in clinical practice [1–8]. Using patient reported outcomes measures (PROMs) to obtain a patient’s self-report of their symptoms across multiple domains in a systematic manner can be a powerful means for understanding an individual’s lived experience of their health. Medical professionals can use PROMs to quickly and efficiently ascertain information about a patient’s symptoms and well-being, and use this information to target areas for intervention. For example, the Patient Reported Outcomes Measurement Information System (PROMIS®) [9] contains a vast array of scales, short forms, and computer adaptive tests (CATs) assessing domains and subdomains [10] of physical function, physical symptoms/medical complications, positive emotions and emotional distress, and social roles, relationships, and participation. Likewise, researchers have undertaken considerable efforts to integrate PROMs into clinical practice [11, 12]. These include implementation of an application programming interface (API) [13] to make PROMIS measures and scores available directly with electronic health record (EHR) systems such as Epic [14].

Clinical use of PROMs is particularly useful in rehabilitation settings. A sudden, traumatic event with potentially lifelong sequelae is unlike other chronic health conditions, such as heart disease or diabetes, which typically have a gradual onset and progressive course. These sudden-onset conditions, such as traumatic brain injury (TBI), stroke, spinal cord injury (SCI), and impaired limb function from traumatic injury or sudden-onset illness, occur in an instant and dramatically change a person’s life in irreversible ways. While there is potential for varying degrees of physical, functional, and cognitive recovery with rehabilitation, individuals who are living with acquired disabilities may experience a wide range of challenging symptoms and functional limitations across physical, emotional, and social domains. To that end, we have taken a holistic view of symptoms across multiple dimensions. Instead of focusing more narrowly on symptoms that are notable by their presence (such as pain and fatigue), we also consider limitations in (or absence of) physical and social function. Taken together, these symptoms greatly impact quality of life (QOL) and present major challenges for clinicians tasked with diagnosing and treating these patients [15–18].

A *symptom cluster* refers to two or more concurrent symptoms that may have a synergistic relationship and/or a shared etiology; in some cases, one symptom will cause changes in a patient’s experience of other related symptoms [19–23]. Symptom clusters research or *symptom science* [24, 25] was originally concentrated in oncology populations [26–57] but has also shown promise for use in cardiac [58–61], gastrointestinal [62], musculoskeletal [63], and mental health [64–73] conditions. The National Institute of Nursing Research (NINR) has been an early and leading proponent of using symptom science to advance clinical care by expanding their intramural program [74] and sponsoring extramural, symptom cluster-specific grant initiatives [75]. There is a growing demand for information on the interplay between co-occurring symptoms, particularly to identify those that share a causal relationship or common underlying cause [76]. Providing clinicians with data-driven information about the interrelationships between symptoms can support their ability to treat multiple symptoms concurrently [77].

Symptom clusters may be identified and implemented from both symptom-oriented (variable-oriented) and person-oriented approaches. Each approach is based on different assumptions concerning the etiological basis [78] and population invariance [79] of symptom clusters, and convergence (or divergence) of results across the two methods can provide important information [20]. Therefore, our mission was two-fold: (1) to determine which symptoms go together using data reduction techniques (e.g., confirmatory factor analysis [CFA]) and creating index or composite scores for higher-order constructs, and (2) to identify patterns of symptoms across these clusters that represent different symptom profiles, such that different treatments or interventions may be indicated for different profiles. However, before determining profile membership, it is first necessary to identify which symptoms must be assessed through a variable-oriented approach. This is the focus of the present manuscript.

While some recent work has identified symptom profiles (groupings of individuals) within rehabilitation populations, the initial step of determining which symptoms cluster together and how to standardize their measurement has not been sufficiently addressed. Notably, Chen et al*.* [80] have combined network and latent profile analysis (LPA) to determine depression-related clusters and trajectory profiles in individuals with stroke, and Breazeale et al*.* [81] have used LPA to identify profiles following limb trauma. In SCI, pain-related clusters and profiles are perhaps the most studied [82, 83], while in TBI profiles are often defined by neurobehavioral [84] and/or psychiatric [85, 86] symptoms. However, while these predominantly person-centered results form a foundation for ongoing work, recent guidance on the symptom science state of the art (i.e., [24]) recommends examining symptom clusters *across* as well as *within* chronic conditions, and emphasizes the need to determine the most important symptoms to measure and the best measures to assess these symptoms [24]. Therefore, we believe that the current literature on symptom clusters in acquired disability populations is limited for two main reasons: (1) for considering symptom clusters only *within* individual populations [87], and (2) by only examining symptoms within a limited number of domains (e.g., physical functioning only), and thus only administering PROMs for a predetermined set of symptoms and not exploring beyond those a priori expectations. Our research seeks to address both limitations.

We believe that exploring symptom clusters across populations and across a wide variety of physical, emotional, and social symptoms will advance understanding of the shared experiences of rehabilitation populations. This manuscript describes our work to identify variable-oriented symptom clusters across the acquired disability populations of SCI, TBI, stroke, and limb injury/illness. For cluster specification, we implemented multi-factor CFA [88] because (a) it directly reduces multivariate data based on patterns of symptom covariation, (b) provides a formal framework for model evaluation and comparison of measurement across multiple groups [78], (c) accounts for measurement error in symptom reporting, and (d) it is the technique we used in our previous work to identify models [89–92] and potential relationships between variables in an a priori fashion [78]. As such, CFA permitted us to identify an optimal number of dimensions possibly underlying a diverse array of outcomes (i.e., PROMs and performance-based cognitive tests) across multiple health domains pertinent to individuals with acquired disability. Secondarily, we attempted to determine whether the dimensions were invariant across injury types and if they exhibited stability over time.

## Methods

### Participants

Participants were adults with a history of sudden-onset acquired disability associated with at least one of four possible diagnoses: (1) TBI, (2) SCI, or (3) stroke, or (4) limb injury or loss due to trauma or sudden-onset illness. Participants were recruited from three sites in the U.S., including two medical rehabilitation centers and an academic university with an active physical therapy clinic. At the rehabilitation centers, potential participants were identified through local research registries and/or medical record review. At the university, potential participants were identified through a research registry of individuals with medically confirmed stroke. At all sites, potential participants were approached by a study team member either in person or by phone, letter, and/or email. Participants were required to be at least 18 years old, have at least one of the four injury diagnoses confirmed via medical record review, and be able to read and understand English. All study procedures were approved by the single IRB of record at the lead site (University of Delaware), and all participants provided informed consent prior to study participation.

### Data collection procedures

Data collection involved (1) abstraction of medical/injury history from participants’ medical and research records, and (2) participant phone interviews. Interviews were conducted by trained research assistants and lasted approximately 2–3 h; participants were permitted to split the interview into more than one session, if needed, as long as the sessions were completed within a short timeframe (≤ 14 days). A subset of participants with each diagnosis completed a second interview 16 months (± 2 months) later. The 16-month timepoint was selected to allow as much time as possible for the expression of any naturally occurring changes so that we could most accurately assess the stability of our cluster solution.

### Measures

Participants completed up to 70 PROMs, as relevant. For example, items related to bowel and bladder functioning were not relevant to most participants, and thus were only administered to participants who endorsed using a bowel or bladder management program; a series of screening questions was used to include only relevant PROMs in each participant’s interview. All participants also completed eight performance-based cognitive tests, several of which are part of the Brief Test of Adult Cognition by Telephone (BTACT) [93]. A complete list of administered measures may be found as Table S1 in the Supplemental Material. Selection of measures for inclusion was informed by: (1) qualitative reanalysis of focus group data from individuals with SCI and TBI [94]; (2) quantitative reanalysis of existing PROM data on individuals with SCI [95, 96], TBI, and limb injury; and by (3) expert input. The inclusion of such a comprehensive set of PROs and performance-based measures was needed to evaluate the full range of potential interrelationships between variables both across and within disability groups. While the analyses presented here evaluate our primary aim, a full set of variables will allow several secondary analyses in the future including initial evaluation of condition specific models that include targeted, population-specific measures. We expect that future researchers can use our results to inform selection of more parsimonious sets of variables for data collection.

Prior to conducting data analyses, a subset of measures was selected for CFA due to limitations on number of variables that can be included in model estimation. Data from all available PROM and cognitive tests were reviewed to determine which subset of variables should be included in the CFA. For the performance-based cognitive tests, we included the three tasks thought to most efficiently measure fluid cognition: Category Fluency, Digit Span Backward, and RAVLT Immediate Recall. In selecting PROMs, we prioritized more general measures representing common symptoms experienced by all four injury groups (e.g., depression, anxiety, pain interference). We omitted measures of symptoms thought of as highly specific to one disability group (e.g., bladder complications, pressure ulcers, satisfaction with orthosis/prosthesis). Finally, we excluded PROMs with strong floor and/or ceiling effects (outcomes with > 40% of individuals at the floor/ceiling were typically excluded; e.g., dyspnea) or those which were highly collinear with other variables (r > 0.85; e.g., Loneliness and Social Isolation). This resulted in a subset of 26 outcome measures being carried forward for the analyses reported herein (23 PROMs and 3 performance-based cognitive tests; see Table 1).

**Table 1:** Outcome measures included in CFA

Measure	Source	# of Items	Metric
*Cognitive health (performance-based)*			
Category fluency	BTACT	1	Total correct
WAIS-III digit span backward	BTACT	16	Total correct
RAVLT immediate recall	BTACT	15	Trials 1–5 correct
*Economic well-being*			
Economic pressure—financial cutbacks	Conger economic pressures scale	29	Raw total
Economic pressure—material needs	Conger economic pressures scale	6	Raw total
Economic quality of life	Economic quality of life (ECON-QOL)	11	Raw total
*Emotional health—Neg affect*			
Anger	PROMIS	7	PROMIS T score
Anxiety	PROMIS	7	PROMIS T score
Depression	PROMIS	8	PROMIS T score
Grief and loss	SCI-QOL, TBI-QOL, LIMB-QOL	10	Raw total
*Emotional health—Pos affect*			
Positive affect and well being	Neuro-QoL	9	Neuro-QoL T score
Resilience	SCI-QOL, TBI-QOL, LIMB-QOL	11	Raw total
*Emotional health—self-image*			
Self esteem	SCI-QOL, TBI-QOL, LIMB-QOL	9	Raw total
*Independence*			
Independence	SCI-QOL, TBI-QOL	12	Raw total
*Physical function*			
Lower extremity function	Neuro-QoL	8	Neuro-QoL T score
Self-care	SCI-FI/C	9	Raw total
*Physical symptoms*			
Fatigue	PROMIS	7	PROMIS T score
Fatigue severity	Fatigue severity scale	9	Raw total
Nociceptive pain quality	PROMIS	5	PROMIS T score
Pain intensity	PROMIS	3	PROMIS T score
Pain interference	PROMIS	8	PROMIS T Score
Sleep Impairment	PROMIS	8	PROMIS T score
*Social health*			
Ability to participate in SRA	Neuro-QoL	8	Neuro-QoL T score
Satisfaction with SRA	Neuro-QoL	8	Neuro-QoL T score
Social isolation	PROMIS	9	PROMIS T score
Stigma	Neuro-QoL	8	Neuro-QoL T score

BTACT = Brief Test of Adult Cognition by Telephone. LIMB-QOL = Limb Injury Measurement Battery for Quality of Life. Neuro-QoL = Quality of Life in Neurological Disorders. PROMIS = Patient-Reported Outcomes Measurement Information System. RAVLT = Rey Auditory Verbal Learning Test. SRA = social roles and activities. TBI-QOL = Traumatic Brain Injury-Quality of Life. WAIS-III = Wechsler Adult Intelligence Scale, Third edition. T-Scores for PROMIS and Neuro-QoL are centered at M = 50 with SD = 10 and normed on the general (PROMIS) or individuals with neurological disorders (Neuro-QoL) populations. All T-Scores were computed using item parameter information from existing or customized short forms (i.e., expected a posteriori scores). All outcomes sourced from ECON-QOL, LIMB-QOL, SCI-QOL, or TBI-QOL were scored as raw total scores as these measures do not have common norm sets; for each measure, raw total scores were calculated as the mean across items × number of item responses

### Data analysis

CFA models were estimated via the *lavaan* package [97] in the R statistical language [98]. All outcomes were continuous variables; therefore, we used robust full-information maximum likelihood (FIML) to estimate all CFA models. Analyses proceeded in three steps. The first step was to determine a global model that provided optimal fit to the data. We used a model comparison approach via CFA with 11 anticipated models developed based on prior work in this area, which informed the specification of candidate models. Prior to analyses, the research team specified 11 candidate models (Table S2), which increased in the number of factors from one to 10. Model fit was quantified primarily through five fit indices, including the Comparative Fit Index (CFI [99]; acceptable fit > 0.90, excellent fit > 0.95), Tucker-Lewis Index (TLI [100]; acceptable fit > 0.90, excellent fit > 0.95), Root Mean Square Error of Approximation (RMSEA [101]; acceptable fit < 0.08, excellent fit < 0.06), and Standardized Root Mean Square Residual (SRMR [102]; acceptable fit < 0.06), as well as the Bayesian Information Criterion (BIC) [103], with lower values indicative of better model fit.

Following the initial evaluation of the 11 models, slight modifications to the optimal/best fitting solution (e.g., allowing split loadings of an observed variable on a latent trait) were specified and evaluated to determine if model fit could be improved through modification indices (i.e., LaGrange multiplier tests). Specifically, the placement of two outcomes, Grief and Loss and Social Isolation, were explicitly tested in the more complex models, given the possibility of these outcomes loading onto more than one factor.

A secondary objective was to examine the measurement invariance of the model selected in step one, to determine whether the factors exhibited the same interpretation across all four injury groups. Following standard invariance testing procedures [104], three models with increasing levels of constraints on model parameters were estimated and compared in terms of model fit. First, a *configural-invariant* model was fit in which the same factor structure was fit within each injury group, with all model parameters freely estimated within groups. Next, a *metric-invariant* model was fit in which factor loadings were constrained to equality across the four groups. Finally, a *scalar-invariant* model was fit in which indicator intercepts were constrained to equality across groups. Models were compared sequentially, and if fit was approximately equivalent (or better) in the more constrained version, invariance constraints were supported. Specifically, decreases in fit (in absolute value) of less than 0.01 in the CFI and RMSEA [105] and 0.03 (0.015) in the SRMR for metric (scalar) invariance [106] were indicative of invariance across groups. Due to wide acknowledgment that invariance may be better conceptualized as approximate rather than exact, we allowed for the possibility of partial measurement invariance [107]. In the event one of the invariance tests failed, we used a *backward search approach* [108] to identify which indicators (and which groups) exhibited non-invariance. The backward search approach involves sequential relaxation of constraints from the more constrained model based on the highest modification index and has shown good performance in simulation studies [108, 109]. Following recommendations for Type I error control [108], we used a modified p-value of 0.01 (i.e., χ^2^ modification index value of 6.635) as the threshold for non-invariance.

For the final objective, we examined the stability of the factor solution over time in the subset of participants who completed the initial and follow-up interviews (n = 224). Given the sample size, we focused on the replication of model fit and parameter estimates obtained at the initial interview with the follow-up data.

## Results

### Participants

The distribution of age, time since injury, gender, race, and ethnicity of the sudden-onset disability sample (N = 755) is shown in Table 2. On average, participants were approximately 50 years old and 10 years removed from their injury, although the stroke group skewed older compared to the other three injury groups and participants in the SCI group were on average about 15 years removed from injury. All injury groups with the exception of stroke were comprised of 70–80% males, with the stroke group showing an approximately even gender split. Race and ethnicity were largely comparable across injury groups, although there were fewer participants reporting Hispanic ethnicity in the stroke group.

**Table 2:** Participant demographic and injury characteristics

	Brain injury (n = 185)	Limb injury (n = 188)	SCI (n = 190)	Stroke (n = 192)	Overall (N = 755)
	M (SD)	M (SD)	M (SD)	M (SD)	M (SD)
Age	43.8 (15.0)	45.0 (13.5)	50.0 (15.7)	64.0 (12.0)	50.8 (16.2)
Time since injury (years)	8.6 (5.3)	9.2 (8.0)	14.8 (12.0)	7.0 (4.1)	9.9 (8.5)
	n (%)	n (%)	n (%)	n (%)	n (%)
*Gender*					
Female	53 (29)	41 (22)	38 (20)	98 (51)	230 (31)
Male	130 (71)	147 (78)	152 (80)	94 (49)	523 (69)
Not Provided	2 (1)	0 (0)	0 (0)	0 (0)	2 (0)
*Race*					
American Indian/Alaskan Native	0 (0)	0 (0)	0 (0)	1 (1)	1 (0)
Asian	7 (4)	0 (0)	5 (3)	7 (4)	19 (3)
Black	32 (17)	26 (14)	39 (21)	51 (27)	148 (20)
Native Hawaiian / Pacific Islander	0 (0)	1 (1)	0 (0.0)	0 (0.0)	1 (0)
White	126 (68)	140 (74)	133 (70)	122 (64)	521 (69)
Multiracial	3 (2)	8 (4)	5 (3)	6 (3)	22 (3)
Other	17 (9)	12 (7)	8 (4)	5 (3)	42 (6)
Not provided	0 (0)	1 (1)	0 (0)	0 (0)	1 (0)
*Ethnicity*					
Hispanic	35 (19)	37 (20)	30 (16)	6 (3)	108 (14)
Non-Hispanic	148 (80)	148 (79)	160 (84)	185 (96)	641 (85)
Not provided	2 (1)	3 (2)	0 (0)	1 (1)	6 (1)

Due to rounding, percentages may not add to 100

### Selection of global model

**Fig. 1:**
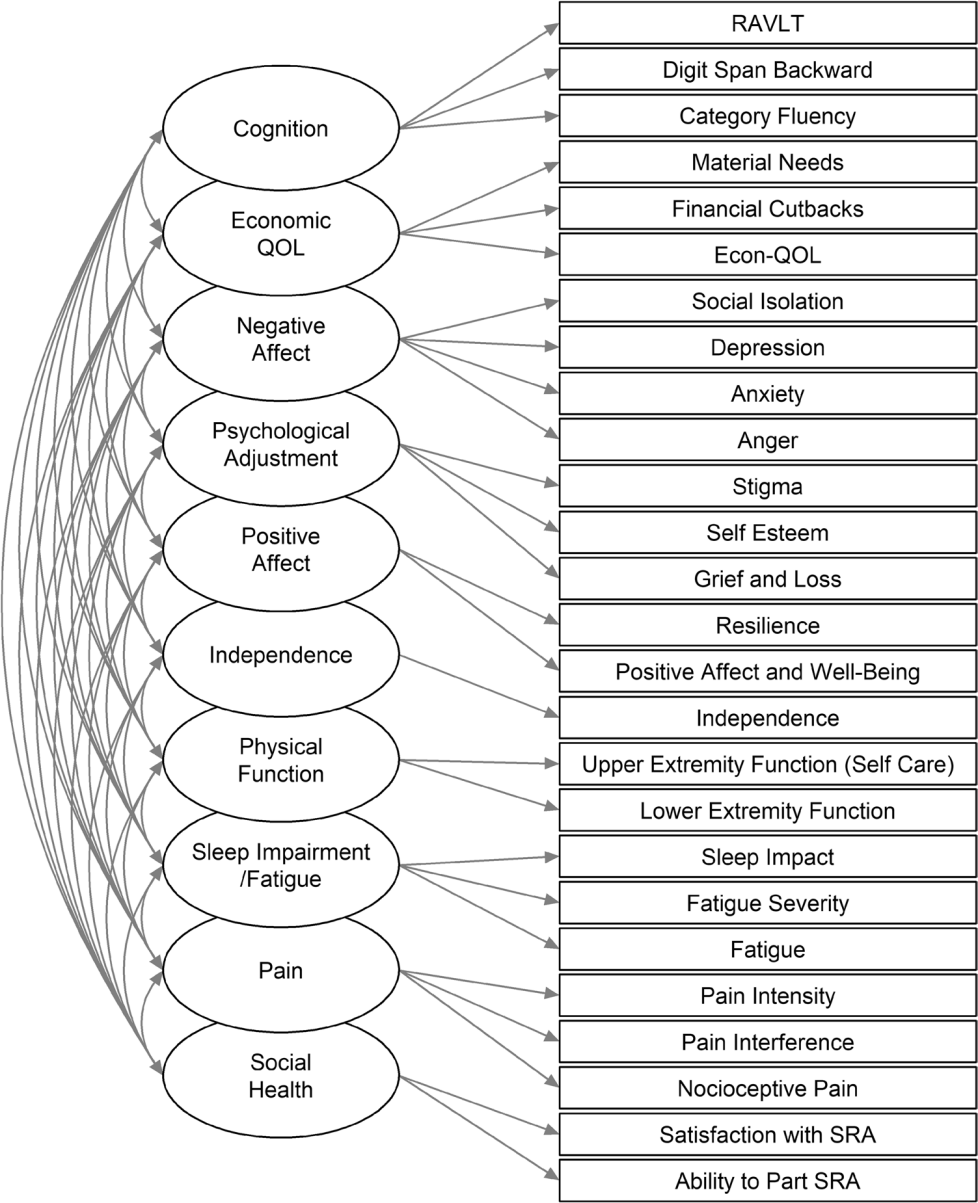
*Path Diagram of Global Model.* In the model shown above, 10 factors are specified to underpin 26 health outcomes across the four injury groups. This model (Model 11) exhibited the closest fit to the data among all alternative specifications and also satisfied conventional thresholds for acceptable global model-to-data fit. Parameter estimates for this model (i.e., factor loading and item intercepts) are reported in Table 4 and factor score correlation estimates are shown in Table 5

Model fit information for global model comparisons is shown in Table 3. In general, model fit improved as the complexity of the factor structure increased. Model 11, which specified 10 factors for the 26 indicators under simple structure (i.e., no dual loadings), exhibited the best model fit among all models, according to each alternative fit index and the BIC. In addition, the RMSEA, CFI, and SRMR satisfied the thresholds for acceptable model fit. Table 4 lists the variables loading on each factor in Model 11 as well as item parameter estimates, and a path diagram of Model 11 is shown in Fig. 1. Standardized factor loadings ranged between 0.54 and 0.94 (in absolute value; item parameter estimates for Model 11 are reported in Table 4). Factor correlations (Table 5) ranged between − 0.80 and 0.89.

**Table 3:** Model fit comparisons of global model

Model	*χ*^*2*^	*df*	*p*	RMSEA	CFI	TLI	SRMR	BIC
1	4705.9	299	< 0.01	0.148	0.622	0.589	0.100	130899.7
2	4192.7	296	< 0.01	0.138	0.672	0.640	0.096	130266.1
3	3955.5	293	< 0.01	0.135	0.692	0.658	0.090	130027.7
4	2942.1	288	< 0.01	0.116	0.775	0.746	0.098	128969.0
5	2167.7	283	< 0.01	0.098	0.841	0.818	0.087	128142.0
6	1901.1	277	< 0.01	0.092	0.863	0.839	0.086	127889.3
7	1140.5	270	< 0.01	0.068	0.927	0.912	0.042	127103.9
8	1070.7	264	< 0.01	0.067	0.932	0.916	0.041	127068.1
9	1040.9	256	< 0.01	0.067	0.934	0.916	0.040	127086.5
10	1019.1	255	< 0.01	0.066	0.936	0.918	0.040	127067.7
11	**875.0**	**255**	** < 0.01**	**0.059**	**0.948**	**0.933**	**0.039**	**126910.6**

Optimal fit values for each index across models are highlighted in bold

**Table 4:** Factor loading and item intercept estimates for selected global model

Measure	Factor loadings				Item Intercepts			
	Est	SE	*p*	Std Est	Est	SE	*p*	Std Est
*Cognition*								
Category fluency	3.24	0.26	0.00	0.57	19.94	0.21	0.00	3.51
Digit span backward	1.35	0.11	0.00	0.54	6.94	0.09	0.00	2.79
RAVLT immediate Recall	6.86	0.49	0.00	0.66	41.68	0.38	0.00	4.02
*Economic QOL*								
Economic QOL	8.39	0.33	0.00	0.89	44.10	0.34	0.00	4.67
Economic Pressure—financial cutbacks	-3.08	0.19	0.00	-0.69	3.64	0.16	0.00	0.81
Economic Pressure—material needs	-4.93	0.20	0.00	-0.85	12.54	0.21	0.00	2.17
*Negative Affect*								
Anger	7.69	0.33	0.00	0.76	48.35	0.37	0.00	4.75
Anxiety	8.22	0.29	0.00	0.82	50.13	0.36	0.00	5.01
Depression	8.90	0.24	0.00	0.91	49.41	0.36	0.00	5.06
Social isolation	7.92	0.29	0.00	0.80	47.20	0.36	0.00	4.75
*Psychological adjustment*								
Grief and Loss	8.62	0.28	0.00	0.85	22.65	0.37	0.00	2.23
Self esteem	-7.34	0.27	0.00	-0.94	37.54	0.29	0.00	4.78
Stigma	6.10	0.27	0.00	0.75	49.00	0.30	0.00	6.03
*Positive Affect*								
Positive affect & well-being	8.76	0.36	0.00	0.87	44.46	0.37	0.00	4.40
Resilience	6.22	0.38	0.00	0.83	39.50	0.27	0.00	5.27
*Independence*								
Independence	6.86	0.23	0.00	0.91	55.51	0.27	0.00	7.37
*Physical function*								
Lower extremity function	5.96	0.25	0.00	0.85	43.28	0.26	0.00	6.17
Upper extremity function (self-care)	12.09	0.28	0.00	1.00	46.14	0.44	0.00	3.82
*Sleep impairment/fatigue*								
Fatigue	8.48	0.30	0.00	0.89	51.14	0.35	0.00	5.36
Fatigue severity	8.26	0.39	0.00	0.66	40.69	0.46	0.00	3.24
Sleep impairment	8.48	0.33	0.00	0.78	49.07	0.40	0.00	4.51
*Pain*								
Nociceptive pain quality	7.71	0.31	0.00	0.77	43.60	0.36	0.00	4.38
Pain interference	8.60	0.24	0.00	0.89	53.14	0.35	0.00	5.48
Pain intensity	10.19	0.28	0.00	0.90	52.51	0.41	0.00	4.64
*Social health*								
Ability to participate in SRA	6.43	0.19	0.00	0.87	47.87	0.27	0.00	6.45
Satisfaction w/SRA	5.59	0.19	0.00	0.91	46.78	0.22	0.00	7.64

**Table 5:** Factor score correlation estimates for selected global model

	Cognition	Economic QOL	Negative affect	Psychological adjustment	Positive affect	Independence	Physical function	Sleep impairment/fatigue	Pain	Social health
Cognition	1.00									
Economic QOL	0.26	1.00								
Negative Affect	-0.07	-0.47	1.00							
Psychological Adjustment	-0.12	-0.44	0.89	1.00						
Positive Affect	0.00	0.35	-0.80	-0.76	1.00					
Independence	0.24	0.35	-0.56	-0.64	0.50	1.00				
Physical Function	0.14	0.19	-0.26	-0.31	0.21	0.69	1.00			
Sleep Impairment/Fatigue	-0.10	-0.36	0.70	0.64	-0.59	-0.40	-0.19	1.00		
Pain	-0.08	-0.27	0.49	0.46	-0.33	-0.31	-0.28	0.49	1.00	
Social Health	0.14	0.43	-0.76	-0.76	0.73	0.71	0.50	-0.67	-0.51	1.00

In Model 11, the *Cognition* and *Economic QOL* factors were comprised of 3 measures each. Outcomes related to emotional health were organized into three dimensions: *Negative Affect*, *Positive Affect*, and *Psychological Adjustment*. Placement of Grief and Loss onto the *Psychological Adjustment* factor (as opposed to Negative Affect) resulted in significant improvement in model fit. *Independence* was optimally placed onto a single-indicated factor, separate from *Physical Function*, which was comprised of Lower and Upper Extremity (Self Care) Function. Two physical symptom dimensions emerged: *Sleep Impairment/Fatigue* and *Pain*, each measured by three measures. Finally, *Social Health* was specified by two indicators, as fit comparisons between Models 9 and 11 suggested that Social Isolation was better located on the Negative Affect factor. Regarding relationships between factors, all 3 emotional health factors (*Negative Affect*, *Positive Affect*, *Psychological Adjustment*) and the *Social Health* factor were strongly correlated (absolute value *r*s = 0.73–0.89; Table 5). *Sleep Impairment/Fatigue* also exhibited strong correlations with these factors (absolute value *r*s = 0.59–0.70) as did Independence (absolute value *r*s = 0.50–0.64), although these 2 factors were only moderately correlated (*r* = − 0.40). *Physical Function* was strongly correlated to *Independence* (*r* = 0.69) and *Social Health* (*r* = 0.50) and weakly related to all other factors. Economic QOL, Cognition, and Pain exhibited no greater than moderate correlation to all other factors (*r*s = − 0.47–0.49). Due to the high correlations between the three emotional health clusters, we subsequently fit a partial second-order model, with *Negative Affect*, *Positive Affect*, and *Psychological Adjustment* specified to load onto a second-order emotional health factor. Fit of this model was largely similar to Model 11: χ2 (269) = 950.1, RMSEA = .061, CFI = .942, TLI = 0.930, SRMR = 0.043, BIC = 126903.0. Due to the complexity of conducting tests of measurement invariance across more than two groups, we proceeded with Model 11; further discussion regarding the emotional health clusters is provided in the Discussion.

### Measurement invariance

To determine whether the dimensions were invariant across injury types, the results for tests of measurement invariance conducted at the baseline timepoint are reported in Table 6. Given the heterogeneous presentation of post-injury physical function challenges across injury types, we hypothesized that invariance of the physical function variables would not be supported. The configural invariance model provided acceptable fit to the data. The addition of factor loading equality constraints across injury groups in the metric invariance model decreased fit; although changes in the CFI, RMSEA, and SRMR values were within the bounds of the previously defined thresholds for invariance tests, inspection of the modification indices identified four factor loading constraints that if lifted would result in significant fit improvements. Of note, factor loadings for both *Physical Function* indicator variables (Lower and Upper Extremity [Self-Care] Function) appeared to differ for TBI compared to the cross-group estimate, and factor loadings for Economic Pressures–Financial Cutbacks and Stigma differed for SCI compared to the other 3 groups. These four constraints were released sequentially; the resulting partial metric invariant model did not exhibit significant loss of fit compared to the configural model [Δ*χ*^2^(45) = 53.8, p = 0.18] and exhibited near identical values of other fit indices. Likewise, the test of scalar invariance did not support equivalence of indicator intercepts across all injury groups; however, the sequential release of several intercept constraints across injury groups resulted in a partial scalar invariant model that exhibited equivalent fit according to alternative fit indices (and more parsimonious according to the BIC), despite the *χ*^2^ difference test reaching significance [Δ*χ*^2^(34) = 49.9, p = 0.04]. Parameter estimates for the partial scalar invariance model are provided in the Supplemental Material (Table S3).

**Table 6:** Measurement invariance test results

Model	Comparison Model	*χ*^*2*^	*df*	*p*	CFI	ΔCFI	RMSEA	ΔRMSEA	SRMR	ΔSRMR	BIC
1. Configural Invariance		1750.6	1020	< 0.01	0.945		0.061		0.053		127763.2
2. Metric Invariance	vs. 1	1888.8	1068	< 0.01	0.937	0.008	0.064	0.003	0.070	0.017	127603.3
3. Partial Metric Invariance	vs. 1	1804.4	1065	< 0.01	0.944	0.001	0.061	0.000	0.063	0.010	127533.9
4. Scalar Invariance	vs. 3	2078.9	1109	< 0.01	0.926	0.018	0.069	0.008	0.066	0.003	127526.1
5. Partial Scalar Invariance	vs. 3	1854.3	1098	< 0.01	0.942	0.002	0.061	0.000	0.062	− 0.001	127364.8

**Fig. 2:**
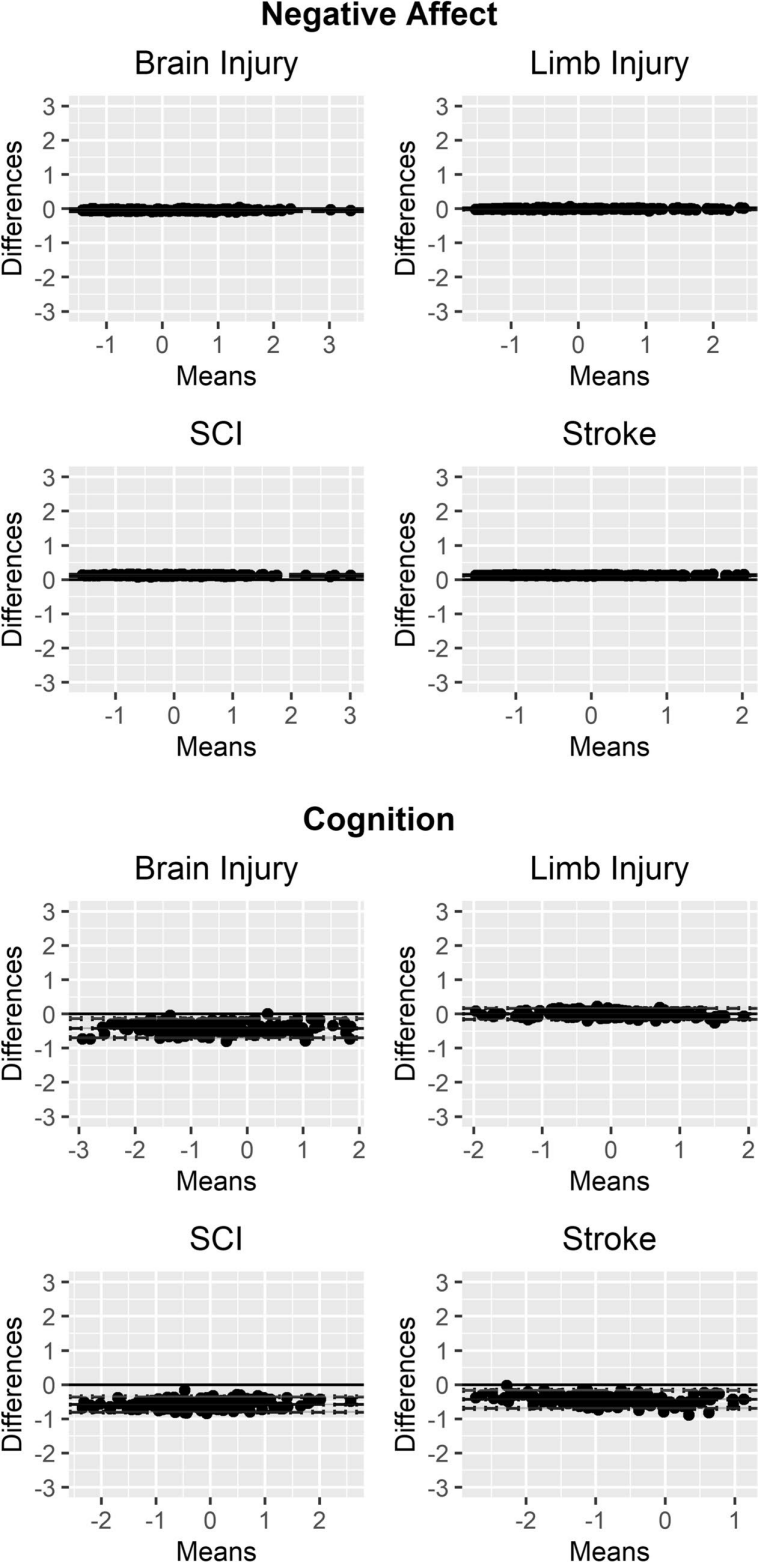

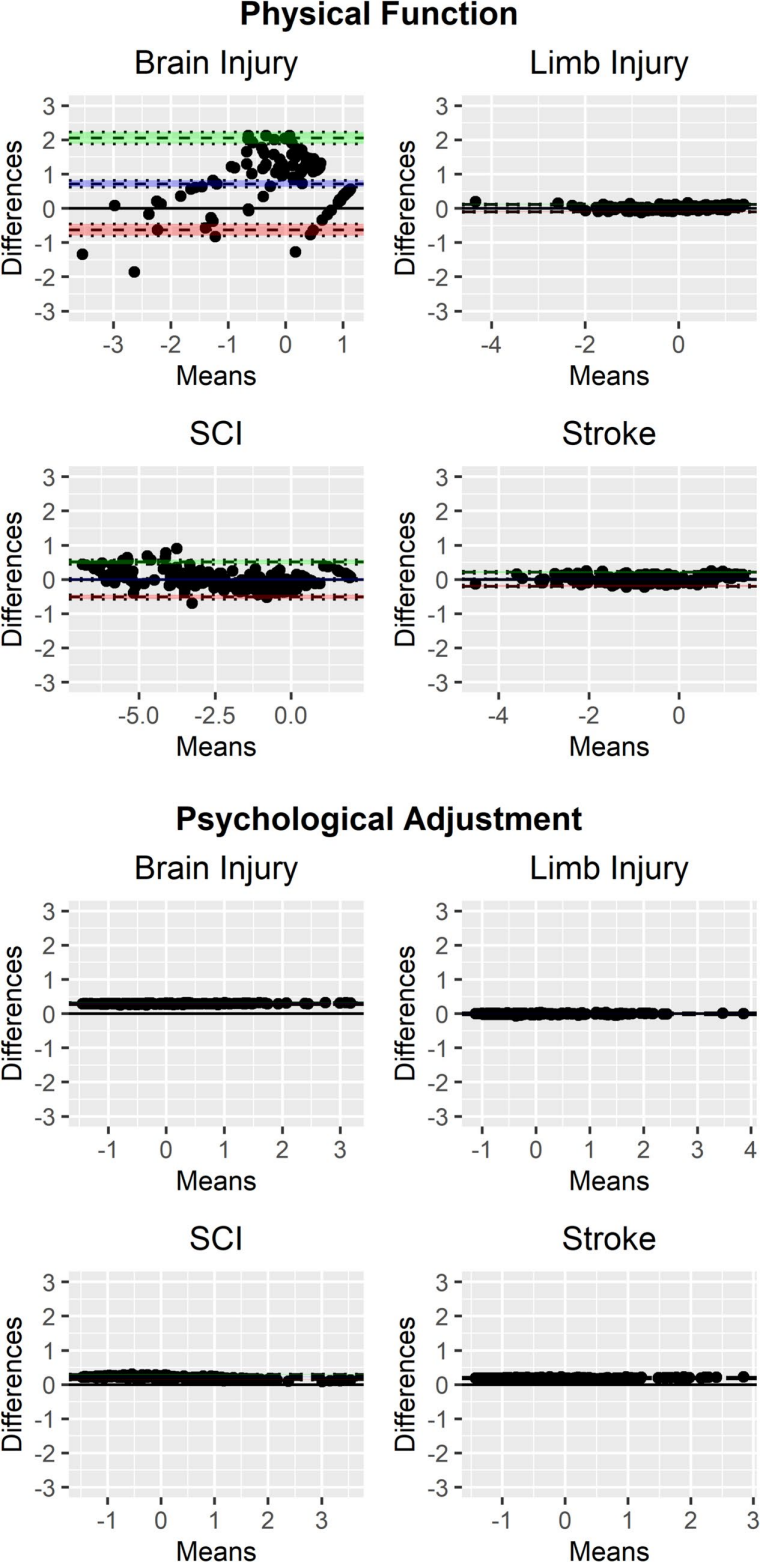
*Bland–Altman Plots for Comparing Full vs. Partial Scalar Invariant Models.* Each Bland–Altman plot shows differences between factors scores generated under a full scalar invariant model—with all factor loadings and intercepts constrained to equality across injury groups—and the partial scalar invariant model. The blue lines represent *bias* (average of all score differences) whereas the green and red lines represent the *limits of agreement* (range in which 95% of all score differences fall). For most factors, score differences were negligible between models (e.g., Negative Affect), although a small degree of bias was observed for Cognition and Psychological Adjustment, and more severe bias for Physical Function

With a small number of exceptions, for approximately half of all model indicators (15 of 26), two intercept estimates were supported in the partial invariance model, suggesting either the estimate for one injury type differed from the common cross-group estimate, or pairs of injury groups exhibited equivalent estimates (e.g., SCI/stroke vs. TBI/limb for Resilience). Despite the presence of some non-invariant factor loadings and intercepts, Bland–Altman plots suggested that differences between factor scores estimated under a full scalar invariant model and the final partial scalar invariant model were negligible for most factors, although bias was greater than 10% for *Cognition*, *Psychological Adjustment*, and, most severely, *Physical Function*. Plots for these factors are shown in Fig. 2 as well as *Negative Affect*, which demonstrated trivial bias and low variance (for comparison). Plots for the remaining factors, all of which demonstrated negligible bias, are provided in Figure S1. Although most instances caused negligible impact on score estimation, physical functioning (e.g., self-care, mobility) differed across conditions, which would impede cross-condition clinical applications and suggests further within-condition analyses are warranted.

### Factor structure stability

Given the reduced follow-up sample size—with only approximately 50 individuals per injury group—we could not formally test longitudinal invariance of the model identified via baseline invariance testing; instead, we focused on the replication of model fit and parameter estimates obtained at the initial interview with the follow-up data. At follow-up, the fit of Model 11 was nearly identical to the fit at baseline: χ^2^ (255) = 430.78, p < 0.01, RMSEA = 0.056, CFI = 0.951, TLI = 0.938, SRMR = 0.050. Differences in standardized factor loadings between time points were small, ranging between − 0.11 and 0.14 (M = 0.01, SD = 0.05). Differences in factor correlations between time points were also small, ranging between − 0.14 and 0.13 (M = 0.01, SD = 0.06). These results suggest stability of the solution over the 14- to 18-month interval.

## Discussion

This study identified a unifying structure for the 26 outcome measures that defined 10 factors across a broad array of physical, emotional, social, and cognitive domains in a combined sample of individuals with SCI, TBI, stroke, or limb injury/illness. This overall structure displayed configural invariance across the four conditions. The 10-factor structure was replicated using the 1-year follow-up data, further supporting the identified solution. Seven of the factors are relatively unique, while the three emotional health factors were more correlated (negative affect, positive affect, and psychological adjustment). Although the improvement in model fit of the CFA suggests that positive emotions, negative emotions, and a more nuanced psychological adjustment factor should be treated as distinct—a finding that echoes studies such as Sherer et al. [92]—the fit of a partial second-order factor model which subsumed these three clusters into a higher-order factor fit equally well. Future studies should further clarify the utility of both approaches, with applications for the time being perhaps best tied to the intended use of the clusters (e.g., parsimony versus descriptive breadth). Overall, however, these findings demonstrate important similarities in the experiences of individuals with these acquired disabilities.

The experience of a sudden-onset disability producing condition can markedly affect nearly every area of a person’s life, from limitations in physical and cognitive function, to changes in emotional well-being and social participation, and impacts on independence and economic quality of life. Although great strides have been made in relevant PRO measurement over the past 20 years, there is not yet a clear way interpret a large number of scores together to get a clear view of an individual’s HRQOL across all of the domains that may be affected by their disability. Previous efforts to obtain a comprehensive understanding of an individual’s current status have done so by administering and scoring each component measure separately. For example, PROMIS profiles (e.g., PROMIS-29) are concatenated sets of brief forms in multiple domains, which each domain score still reported separately.^1^

We believe what has been missing is a set of reliable index or composite scores to distill the information from multiple measures into a holistic view of one’s HRQOL. While the current assessment battery, consisting of 23 PROs and 3 performance-based measures, may be prohibitively long for most applications, there exists tremendous potential to use these clusters or factors to flag areas of concern (for example, using a handful of indicator variables) to monitor or prevent long-term comorbidities. Flagged areas could then be examined in more depth (for example, with follow-up assessment) to inform interdisciplinary treatment planning. It is clear, though, that future work is needed to fully prepare our cluster solution for clinical implementation.

### Limitations and future directions

Participant-related limitations of the study that may limit the generalizability of the results include the requirement that participants speak English and potential selection bias associated with recruiting from research registries. A significant measurement-related limitation is that this study employed more generic or general measures of symptoms and psychosocial issues that are relevant across multiple conditions. Symptoms that are experienced by a specific population—such as neurogenic bowel and/or bladder for individuals with SCI—were not included in the analyses that are presented here. Thus, the models presented here may omit some clinical information that would be relevant to treatment strategies. Moreover, some of the included measures have not been co-normed and therefore it was necessary to use raw scores in analyses; further efforts to standardize metrics across these and other sudden-onset disabling conditions, particularly for outcomes pertinent to these populations (e.g., Independence), is warranted and will further advance interpretation of the clusters.

We found that there was a common factor structure of HRQOL, and the general domain cluster score variables appear to measure the same construct across groups (e.g., our test of configural invariance). However, we also found specific instances of non-invariance. Although most instances caused negligible impact on score estimation, physical functioning (e.g., self-care, fine-motor, mobility) seems to differ across conditions, which suggests further within-condition analyses are warranted.

Targets for further investigation include replicating in non-English-speaking samples, application of the identified cluster solution in intervention studies, and association of clusters with physiological and/or biological markers. Finally, given the potential time- and resource-related obstacles to clinical implementation of such a comprehensive set of outcomes measures, we recommend that future work should be conducted to evaluate the incremental validity and cost-benefits analysis of including such a comprehensive battery of measures [110] and explore opportunities for real-world implementation.

## Conclusion

This study has identified 10 symptom clusters that represent target areas for clinical monitoring and potential intervention. The factor solution appears robust; these results confirm prior work and were replicated in long-term follow-up. The overall 10-factor structure appears invariant across conditions, although the inter-relationships between symptoms may vary within groups. Utilizing symptom clusters in clinical practice, through tools such as cluster-based index scores, could potentially improve understanding of patients’ lived experience of symptoms following sudden-onset acquired disability, which is critical to improving treatments and ultimately, HRQOL outcomes.

## Supplementary Information

Below is the link to the electronic supplementary material.

Supplementary file1 (DOCX 419 KB)

## Data Availability

No datasets were generated or analysed during the current study.

## Abbreviations

BIC: Bayesian information criterion
CAT: Computer adaptive test
CFA: Confirmatory factor analysis
CFI: Comparative fit index
FIML: Full-information maximum likelihood
HRQOL: Health-related quality of life
LIMB-QOL: Limb injury measurement battery for quality of life
LPA: Latent profile analysis
Neuro-QoL: Quality of life in neurological disorders (measurement system)
NINR: National Institute of Nursing Research
PROMs: Patient reported outcome measures
PROMIS: Patient reported outcomes measurement information system
QOL: Quality of life
RMSEA: Root mean square error of approximation
SCI: Spinal cord injury
SCI-QOL: Spinal cord injury-quality of life (measurement system)
SRMR: Standardized root mean square residual
TBI: Traumatic brain injury
TBI-QOL: Traumatic brain injury-quality of life (measurement system)
TLI: Tucker-Lewis index

## Footnotes

1 PROMIS profiles also yield physical and mental health summary scores as well as domain-specific scores for some applications.
