# Supplementary material for: Using confirmatory factor analysis to identify symptom clusters in individuals with acquired disabilities
Source: Qual Life Res. 2026 Apr 1;35(5):114. doi: 10.1007/s11136-026-04220-0 (PMC13043617; doi:10.1007/s11136-026-04220-0)
Supplement: Supplementary file 1 — Supplementary file1 (DOCX 419 KB) [file 11136_2026_4220_MOESM1_ESM.docx]

Supplementary Material

**Title:** Using Confirmatory Factor Analysis to Identify Symptom Clusters in Individuals with Acquired Disabilities

***Journal:*** *Quality of Life Research*

**Authors:**

David S. Tulsky, Ph.D., University of Delaware, dtulsky@udel.edu

Aaron J. Boulton, Ph.D.

Pamela A. Kisala, M.A.

Callie E. Tyner, Ph.D.

Ryan Pohlig, Ph.D.

Nancy D. Chiaravalloti, Ph.D.

Jerry Slotkin, Ph.D.

Heather B. Taylor, Ph.D.

Mark Sherer, Ph.D.

This supplement provides additional detail regarding the measures, models, and findings reported in the main article.

In Table S1, a list of all patient-reported outcome measures (PROM) and performance-based cognitive tests administered to participants in the study is provided. As noted in the main article, measures were selected for inclusion in analyses through a rigorous process involving reanalysis of existing qualitative and quantitative data and expert review. Measures selected covered medical symptoms, functional and psychosocial outcomes, and quality of life endpoints considered common or relevant to all four injury subgroups included in the study.

In Table S2, specifications for the 11 candidate models for the global (cross-condition) confirmatory factor analysis (CFA) are provided.

In Table S3, parameter estimates for the partial scalar invariant model are shown.

In Figure S1, Bland-Altman plots for factors that were not reported in the main manuscript are presented.

Table S1. *Outcome Measures Administered in Baseline and Follow-up Field Testing*

| **Category/Measure** | **Source** | **Used in CFA?** |
| --- | --- | --- |
| **Cognitive Health (Performance-Based)** | | |
| Backward Counting | BTACT |  |
| Category Fluency | BTACT | X |
| WAIS-III Digit Span Backward | WAIS-III/BTACT | X |
| WAIS-III Digit Span Forward | WAIS-III |  |
| Letter Fluency | COWAT |  |
| Number Series | BTACT |  |
| Oral Trail Making Test | Oral Trail Making Test |  |
| RAVLT Immediate Recall | BTACT | X |
| **Cognitive Health (Self-Report)** | | |
| Cognition – General | Neuro-QoL |  |
| Executive Function | Neuro-QoL |  |
| **Economic Well-Being** | | |
| Economic Pressure – Financial Cutbacks | CEPS | X |
| Economic Pressure – Make Ends Meet | CEPS |  |
| Economic Pressure – Material Needs | CEPS | X |
| Economic Quality of Life (ECON-QOL) | ECON-QOL | X |
| Vocational Impact | LIMB-QOL |  |
| **Emotional Health – Neg Affect** | | |
| Anger | TBI-QOL | X |
| Anxiety | PROMIS | X |
| Depression | PROMIS | X |
| Grief and Loss | SCI-QOL, TBI-QOL, LIMB-QOL | X |
| Perceived Stress | NIHTB |  |
| Posttraumatic Stress Disorder (PTSD) Symptoms | PC-PTSD-5 |  |
| Trauma | SCI-QOL |  |
| **Emotional Health – Pos Affect** | | |
| Future Outlook | LIMB-QOL |  |
| Positive Affect and Well Being | Neuro-QoL | X |
| Resilience | SCI-QOL, TBI-QOL, LIMB-QOL | X |
| **Emotional Health – Self-Efficacy** | | |
| General Self Efficacy | PROMIS |  |
| Health-Related Self Efficacy | LIMB-QOL |  |
| Self-Efficacy for Managing Daily Activities | PROMIS |  |
| Self-Efficacy for Managing Emotions | PROMIS |  |
| Self-Efficacy for Managing Symptoms | PROMIS |  |
| **Emotional Health – Self-Image** | | |
| Body Image | LIMB-QOL |  |
| Satisfaction with Physical Abilities/Fitness/Athleticism | LIMB-QOL |  |
| Self-Esteem | SCI-QOL, TBI-QOL, LIMB-QOL | X |
| Weight Satisfaction | LIMB-QOL |  |
| **Independence** | | |
| Craig Handicap Assessment and Reporting Technique (CHART) short form | CHART |  |
| Independence | SCI-QOL, TBI-QOL | X |
| Katz Activities of Daily Living (ADL) | Katz ADL |  |
| Part-O Out and About | Part-O |  |
| **Physical Function** | | |
| Ambulation | SCI-FI/C |  |
| Basic Mobility | SCI-FI/C |  |
| Fine Motor | SCI-FI/C |  |
| Lower Extremity Function | Neuro-QoL | X |
| Manual Wheelchair Mobility | SCI-FI/AT |  |
| Physical Function | PROMIS |  |
| Physical Function with Mobility Aid | PROMIS |  |
| Power Wheelchair Mobility | SCI-FI/AT |  |
| Satisfaction with Orthosis/Prosthesis | LIMB-QOL |  |
| SCI-FI A/T Measures | SCI-FI/AT |  |
| Self-Care | SCI-FI/C | X |
| Upper Extremity | Neuro-QoL |  |
| **Physical Symptoms** | | |
| Bladder Complications | SCI-QOL |  |
| Bladder Management Difficulties | SCI-QOL |  |
| Bowel Management Difficulties | SCI-QOL |  |
| Chronic Pain | Graded Chronic Pain Scale |  |
| Dyspnea Functional Limitations | PROMIS |  |
| Dyspnea Severity | PROMIS |  |
| Fatigue | PROMIS | X |
| Fatigue Severity | Fatigue Severity Scale | X |
| Gastrointestinal – Incontinence | PROMIS |  |
| Gastrointestinal – Nausea | PROMIS |  |
| Headache Impact | HIT-6 |  |
| Headache Pain | TBI-QOL |  |
| Neuropathic Pain Quality | PROMIS |  |
| Nociceptive Pain Quality | PROMIS | X |
| Pain Intensity | PROMIS | X |
| Pain Interference | PROMIS | X |
| Pressure Ulcers | SCI-QOL |  |
| Respiratory Impairment | SCI-QOL |  |
| Sleep Disturbance | PROMIS |  |
| Sleep Impairment | PROMIS | X |
| Stiffness Impact | ASCQ-Me |  |
| **Social Health** | | |
| Ability to Participate in SRA | Neuro-QoL | X |
| Emotional Support | NIHTB |  |
| Instrumental Support | NIHTB |  |
| Loneliness | NIHTB |  |
| Satisfaction with SRA | Neuro-QoL | X |
| Social Isolation | PROMIS | X |
| Stigma | Neuro-QoL | X |

*Notes*. ASCQ-Me = Adult Sickle Quality of Life Measurement Information System. BTACT = Brief Test of Adult Cognition by Telephone. CEPS = Conger Economic Pressures Scale. CFA = Confirmatory factor analysis. COWAT = Controlled Oral Word Association Test. ECON-QOL = Economic Quality of Life. HIT6 = Headache Impact Test-6. LIMB-QOL = Limb Injury Measurement Battery for Quality of Life. NIHTB = NIH Toolbox. Neuro-QoL = Quality of Life in Neurological Disorders. Part-O = Participation Assessment with Recombined Tools-Objective. PROMIS = Patient-Reported Outcomes Measurement Information System. PC-PTSD-5 = Primary Care PTSD Screen for Diagnostic and Statistical Manual-5. RAVLT = Rey Auditory Verbal Learning Test. SCI-FI/C, SCI-FI/AT = Spinal Cord Injury Functional Index – Capacity or Assistive Technology. SCI-QOL = Spinal Cord Injury-Quality of Life. SRA = social roles and activities. TBI-QOL = Traumatic Brain Injury-Quality of Life. WAIS-III = Wechsler Adult Intelligence Scale, Third edition.

*Table S2. Candidate Models for Global CFA Model*

| Measure | M1 | M2 | M3 | M4 | M5 | M6 | M7 | M8 | M9 | M10 | M11 |
| --- | --- | --- | --- | --- | --- | --- | --- | --- | --- | --- | --- |

| Category Fluency | GEN | PSY | COG | COG | COG | COG | COG | COG | COG | COG | COG |
| --- | --- | --- | --- | --- | --- | --- | --- | --- | --- | --- | --- |
| Digit Span Backward | GEN | PSY | COG | COG | COG | COG | COG | COG | COG | COG | COG |
| RAVLT Immediate Recall | GEN | PSY | COG | COG | COG | COG | COG | COG | COG | COG | COG |

| Economic Quality of Life | GEN | SOC | SOC | SOC | ECON | ECON | ECON | ECON | ECON | ECON | ECON |
| --- | --- | --- | --- | --- | --- | --- | --- | --- | --- | --- | --- |
| Economic Pressure - Financial Cutbacks | GEN | SOC | SOC | SOC | ECON | ECON | ECON | ECON | ECON | ECON | ECON |
| Economic Pressure - Material Needs | GEN | SOC | SOC | SOC | ECON | ECON | ECON | ECON | ECON | ECON | ECON |

| Anger | GEN | PSY | EMO | EMO | EMO | NEG | NEG | NEG | NEG | NEG | NEG |
| --- | --- | --- | --- | --- | --- | --- | --- | --- | --- | --- | --- |
| Anxiety | GEN | PSY | EMO | EMO | EMO | NEG | NEG | NEG | NEG | NEG | NEG |
| Depression | GEN | PSY | EMO | EMO | EMO | NEG | NEG | NEG | NEG | NEG | NEG |
| Social Isolation | GEN | PSY | EMO | EMO | EMO | NEG | NEG | NEG | ISO | NEG | NEG |
| Grief and Loss | GEN | PSY | EMO | EMO | EMO | NEG | NEG | NEG | NEG | NEG | ADJ |
| Self-Esteem | GEN | PSY | EMO | EMO | EMO | NEG | NEG | NEG | NEG | ADJ | ADJ |
| Stigma | GEN | PSY | EMO | EMO | EMO | NEG | NEG | NEG | NEG | ADJ | ADJ |

| Positive Affect & Well-Being | GEN | PSY | EMO | EMO | EMO | POS | POS | POS | POS | POS | POS |
| --- | --- | --- | --- | --- | --- | --- | --- | --- | --- | --- | --- |
| Resilience | GEN | PSY | EMO | EMO | EMO | POS | POS | POS | POS | POS | POS |
| Independence | GEN | PHY | PHY | PHY/SOC | PHY/SOC | PHY/SOC | PHY/SOC | IND | IND | IND | IND |
| Lower Extremity Function | GEN | PHY | PHY | PHY | PHY | PHY | PHY | PHY | PHY | PHY | PHY |
| Upper Extremity Function (Self-Care) | GEN | PHY | PHY | PHY | PHY | PHY | PHY | PHY | PHY | PHY | PHY |
| Fatigue | GEN | PHY | PHY | MED | MED | MED | SLP/FAT | SLP/FAT | SLP/FAT | SLP/FAT | SLP/FAT |
| Fatigue Severity | GEN | PHY | PHY | MED | MED | MED | SLP/FAT | SLP/FAT | SLP/FAT | SLP/FAT | SLP/FAT |
| Sleep Impairment | GEN | PHY | PHY | MED | MED | MED | SLP/FAT | SLP/FAT | SLP/FAT | SLP/FAT | SLP/FAT |
| Nociceptive Pain Quality | GEN | PHY | PHY | MED | MED | MED | PAIN | PAIN | PAIN | PAIN | PAIN |
| Pain Interference | GEN | PHY | PHY | MED | MED | MED | PAIN | PAIN | PAIN | PAIN | PAIN |
| Pain Intensity | GEN | PHY | PHY | MED | MED | MED | PAIN | PAIN | PAIN | PAIN | PAIN |
| Ability to Participate in SRA | GEN | SOC | SOC | SOC | SOC | SOC | SOC | SOC | SOC | SOC | SOC |
| Satisfaction w/SRA | GEN | SOC | SOC | SOC | SOC | SOC | SOC | SOC | SOC | SOC | SOC |

*Note*. ADJ = Psychological Adjustment. COG = Cognition. ECON = Economic QOL. EMO = Emotional Health. FAT = Fatigue. GEN = General Health. IND = Independence. MED = Physical Symptoms. PHY = Physical Health. PHY-FUN = Physical Function. PSY = Psychological Health. SLP = Sleep Impairments. SOC = Social Health. NEG = Negative Affect. POS = Positive Affect.

Table S3. *Factor Loading and Item Intercept Estimates for Partial Scalar Invariant Model*

|  | Factor Loading Estimates (Standard Error) | | | | Intercept Estimates (Standard Error) | | | |
| --- | --- | --- | --- | --- | --- | --- | --- | --- |
| Measure | TBI | Limb | SCI | Stroke | TBI | Limb | SCI | Stroke |
| *Cognition* |  |  |  |  |  |  |  |  |
| Category Fluency | 2.59 (0.58) | 2.59 (0.46) | 2.59 (0.53) | 2.59 (0.53) | 19.63 (3.55) | **23.21 (4.16)** | 19.63 (3.82) | 19.63 (3.90) |
| Digit Span Backward | 1.21 (0.60) | 1.21 (0.52) | 1.21 (0.49) | 1.21 (0.52) | 7.21 (2.87) | 7.21 (3.07) | 7.21 (2.78) | 7.21 (2.99) |
| RAVLT Immediate Recall | 6.41 (0.69) | 6.41 (0.64) | 6.41 (0.72) | 6.41 (0.66) | 43.76 (3.80) | 43.76 (4.34) | **41.28 (4.38)** | 43.76 (4.36) |
| *Economic QOL* |  |  |  |  |  |  |  |  |
| Economic QOL | 8.10 (0.85) | 8.10 (0.91) | 8.10 (0.90) | 8.10 (0.88) | 45.67 (4.84) | 45.67 (5.11) | 45.67 (4.66) | 45.67 (5.05) |
| Economic Pressure - Financial Cutbacks | -3.48 (-0.74) | -3.48 (-0.73) | **-1.97 (-0.55)** | -3.48 (-0.78) | 2.54 (0.54) | **3.56 (0.74)** | 2.54 (0.65) | **3.56 (0.81)** |
| Economic Pressure - Material Needs | -4.81 (-0.80) | -4.81 (-0.85) | -4.81 (-0.89) | -4.81 (-0.86) | 11.65 (1.96) | 11.65 (2.04) | 11.65 (1.98) | 11.65 (2.12) |
| *Negative Affect* |  |  |  |  |  |  |  |  |
| Anger | 8.12 (0.77) | 8.12 (0.81) | 8.12 (0.71) | 8.12 (0.75) | 48.85 (4.45) | 48.85 (4.88) | 48.85 (4.76) | 48.85 (5.30) |
| Anxiety | 8.62 (0.84) | 8.62 (0.85) | 8.62 (0.78) | 8.62 (0.81) | **49.61 (4.61)** | 51.02 (5.04) | 51.02 (5.17) | 51.02 (5.64) |
| Depression | 9.40 (0.95) | 9.40 (0.93) | 9.40 (0.89) | 9.40 (0.90) | 48.70 (4.68) | 48.70 (4.81) | **51.22 (5.43)** | **51.22 (5.80)** |
| Social Isolation | 8.19 (0.83) | 8.19 (0.78) | 8.19 (0.78) | 8.19 (0.76) | 47.73 (4.61) | 47.73 (4.52) | 47.73 (5.08) | 47.73 (5.24) |
| *Psychological Adjustment* |  |  |  |  |  |  |  |  |
| Grief and Loss | 8.44 (0.85) | 8.44 (0.87) | 8.44 (0.88) | 8.44 (0.82) | 24.95 (2.40) | **20.22 (2.09)** | 24.95 (2.41) | 24.95 (2.56) |
| Self-Esteem | -7.20 (-0.93) | -7.20 (-0.97) | -7.20 (-0.94) | -7.20 (-0.92) | 35.46 (4.37) | ***37.60 (5.07)*** | **36.58 (4.44)** | **36.58 (4.89)** |
| Stigma | 6.35 (0.77) | 6.35 (0.78) | **5.05 (0.70)** | 6.35 (0.77) | 49.62 (5.72) | 49.62 (6.12) | **50.39 (6.51)** | 49.62 (6.34) |
| *Positive Affect* |  |  |  |  |  |  |  |  |
| Positive Affect & Well-Being | 6.55 (0.93) | 6.55 (0.89) | 6.55 (0.92) | 6.55 (0.90) | 55.85 (7.06) | 55.85 (7.59) | 55.85 (7.62) | 55.85 (7.58) |
| Resilience | 5.66 (0.87) | 5.66 (0.83) | 5.66 (0.86) | 5.66 (0.84) | 42.87 (5.86) | 42.87 (6.25) | **44.29 (6.54)** | **44.29 (6.51)** |
| *Independence* |  |  |  |  |  |  |  |  |
| Independence | 10.34 (1.00) | 10.34 (1.00) | 10.34 (1.00) | 10.34 (1.00) | 49.52 (4.43) | 49.52 (4.79) | 49.52 (3.72) | 49.52 (4.14) |
| *Physical Function* |  |  |  |  |  |  |  |  |
| Lower Extremity Function | **8.51 (1.00)** | 4.27 (0.56) | 4.27 (0.92) | 4.27 (0.74) | **50.92 (5.96)** | 46.07 (6.06) | 46.07 (4.07) | 46.07 (6.18) |
| Upper Extremity Function (Self-Care) | **2.45 (0.69)** | 3.57 (0.66) | 3.57 (0.87) | 3.57 (0.81) | **43.21 (12.13)** | 41.33 (7.59) | 41.33 (4.09) | 41.33 (7.25) |
| *Sleep Impairment/Fatigue* |  |  |  |  |  |  |  |  |
| Fatigue | 7.69 (0.93) | 7.69 (0.86) | 7.69 (0.89) | 7.69 (0.91) | 51.35 (5.12) | 51.35 (5.71) | 51.35 (5.85) | 51.35 (5.06) |
| Fatigue Severity | 7.46 (0.72) | 7.46 (0.61) | 7.46 (0.64) | 7.46 (0.69) | 40.18 (3.18) | 40.18 (3.28) | 40.18 (3.38) | **43.16 (3.32)** |
| Sleep Impairment | 7.54 (0.80) | 7.54 (0.75) | 7.54 (0.74) | 7.54 (0.80) | 50.03 (4.35) | 50.03 (4.97) | 50.03 (4.83) | **47.13 (4.18)** |
| *Pain* |  |  |  |  |  |  |  |  |
| Nociceptive Pain Quality | 7.37 (0.81) | 7.37 (0.70) | 7.37 (0.80) | 7.37 (0.83) | 45.41 (4.72) | 45.41 (4.32) | 45.41 (4.56) | 45.41 (4.65) |
| Pain Interference | 8.00 (0.89) | 8.00 (0.88) | 8.00 (0.87) | 8.00 (0.89) | 55.19 (5.84) | 55.19 (6.10) | 55.19 (5.57) | 55.19 (5.63) |
| Pain Intensity | 9.39 (0.90) | 9.39 (0.87) | 9.39 (0.91) | 9.39 (0.91) | 54.38 (4.95) | 54.38 (5.01) | **56.50 (5.07)** | 54.38 (4.81) |
| *Social Health* |  |  |  |  |  |  |  |  |
| Ability to Participate in SRA | 6.28 (0.86) | 6.28 (0.92) | 6.28 (0.86) | 6.28 (0.82) | 47.72 (6.18) | 47.72 (7.01) | 47.72 (6.30) | 47.72 (6.47) |
| Satisfaction w/SRA | 5.48 (0.91) | 5.48 (0.92) | 5.48 (0.93) | 5.48 (0.91) | 46.96 (7.38) | 46.96 (7.84) | 46.96 (7.66) | **45.90 (7.87)** |

*Notes*. Because standardization of estimates in CFA is a post-estimation procedure, estimates constrained to equality are not expected to be equivalent following standardization. Instances of non-invariance are highlighted in bold. For Self-Esteem, the intercept estimate for the TBI group differed from all other groups, the estimate for the Limb group differed from all other groups (bold italic), and the estimate for SCI and Stroke were constrained to equality (bold).

Table S4. *Percentage of Sample at Minimum / Maximum Values (i.e., Floor/Ceiling)*

|  | Brain  Injury | | Limb  Injury | | | SCI | | Stroke | |
| --- | --- | --- | --- | --- | --- | --- | --- | --- | --- |
| Measure | % at Min | % at Max | | % at Min | % at Max | % at Min | % at Max | % at Min | % at Max |
| Category Fluency | 0.5 | 1.1 | | 1.0 | 1.0 | 0.5 | 0.5 | 0.5 | 1.0 |
| WAIS-III Digit Span Backward | 1.1 | 1.6 | | 1.0 | 1.0 | 2.0 | 2.0 | 3.6 | 0.5 |
| RAVLT Immediate Recall | 0.5 | 0.5 | | 0.5 | 0.5 | 0.5 | 0.5 | 0.5 | 1.0 |
| Economic Pressure – Financial Cutbacks | 29.0 | 1.1 | | 35.3 | 0.5 | 42.2 | 0.5 | 37.6 | 0.5 |
| Economic Pressure – Material Needs | 13.5 | 1.6 | | 27.4 | 1.0 | 17.1 | 1.5 | 25.6 | 0.5 |
| Economic Quality of Life | 1.6 | 10.8 | | 0.5 | 13.9 | 0.5 | 10.0 | 1.0 | 13.7 |
| Anger | 23.7 | 0.5 | | 28.1 | 0.5 | 27.9 | 0.5 | 35.5 | 0.5 |
| Anxiety | 21.0 | 1.1 | | 20.3 | 0.5 | 21.6 | 0.5 | 23.4 | 0.5 |
| Depression | 27.4 | 0.5 | | 29.1 | 0.5 | 21.4 | 1.0 | 26.4 | 1.0 |
| Grief and Loss | 8.6 | 1.6 | | 13.8 | 0.5 | 8.9 | 1.0 | 11.1 | 1.0 |
| Positive Affect and Well Being | 0.5 | 11.8 | | 0.5 | 11.3 | 0.5 | 10.4 | 0.5 | 16.2 |
| Resilience | 1.1 | 4.8 | | 0.5 | 3.0 | 0.5 | 5.4 | 0.5 | 4.5 |
| Self Esteem | 1.1 | 22.0 | | 0.5 | 15.3 | 1.5 | 23.3 | 0.5 | 24.7 |
| Independence | 0.5 | 18.3 | | 0.5 | 14.3 | 0.5 | 5.9 | 0.5 | 12.1 |
| Lower Extremity Function | 0.5 | 50.5 | | 0.5 | 18.7 | 6.9 | 4.0 | 0.5 | 12.8 |
| Self-Care | 0.5 | 61.3 | | 0.5 | 34.5 | 4.5 | 21.3 | 0.5 | 17.3 |
| Fatigue | 2.7 | 0.5 | | 0.5 | 0.5 | 2.0 | 0.5 | 2.5 | 0.5 |
| Fatigue Severity | 1.1 | 0.5 | | 1.0 | 0.5 | 0.5 | 0.5 | 1.5 | 0.5 |
| Nociceptive Pain Quality | 30.1 | 1.6 | | 9.9 | 2.0 | 19.0 | 1.5 | 29.6 | 0.5 |
| Pain Intensity | 36.0 | 1.1 | | 19.7 | 2.5 | 25.7 | 3.0 | 33.5 | 0.5 |
| Pain Interference | 30.1 | 0.5 | | 14.3 | 0.5 | 15.8 | 1.5 | 28.9 | 1.5 |
| Sleep Impairment | 7.5 | 0.5 | | 5.0 | 0.5 | 7.5 | 0.5 | 10.7 | 0.5 |
| Ability to Participate in SRA | 0.5 | 23.7 | | 0.5 | 13.8 | 1.0 | 16.8 | 0.5 | 21.2 |
| Satisfaction with SRA | 1.1 | 12.9 | | 0.5 | 10.8 | 1.5 | 7.4 | 0.5 | 8.1 |
| Social Isolation | 14.5 | 1.1 | | 19.1 | 0.5 | 19.4 | 0.5 | 23.4 | 1.0 |
| Stigma | 23.2 | 2.7 | | 23.2 | 0.5 | 23.2 | 3.5 | 32.8 | 0.5 |


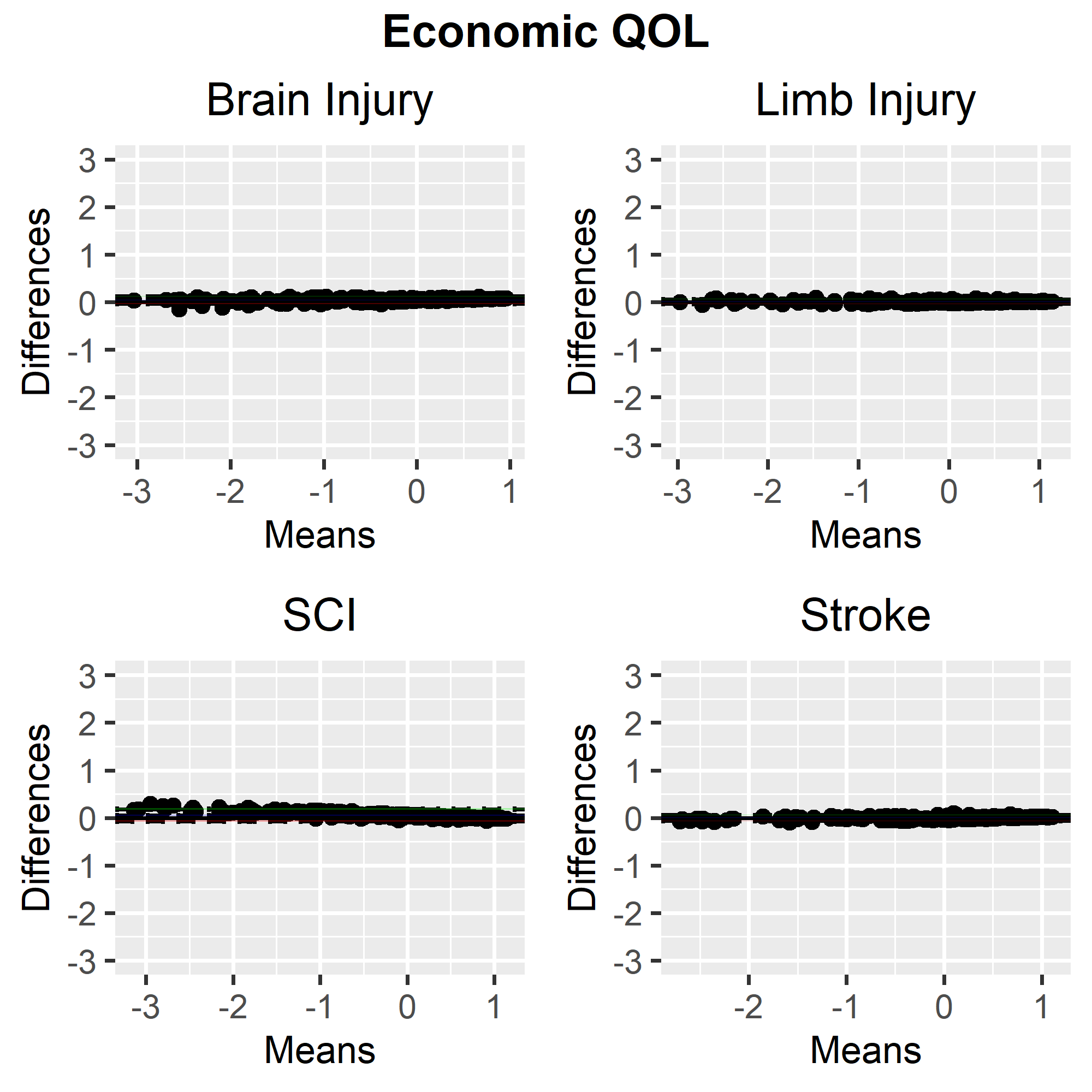


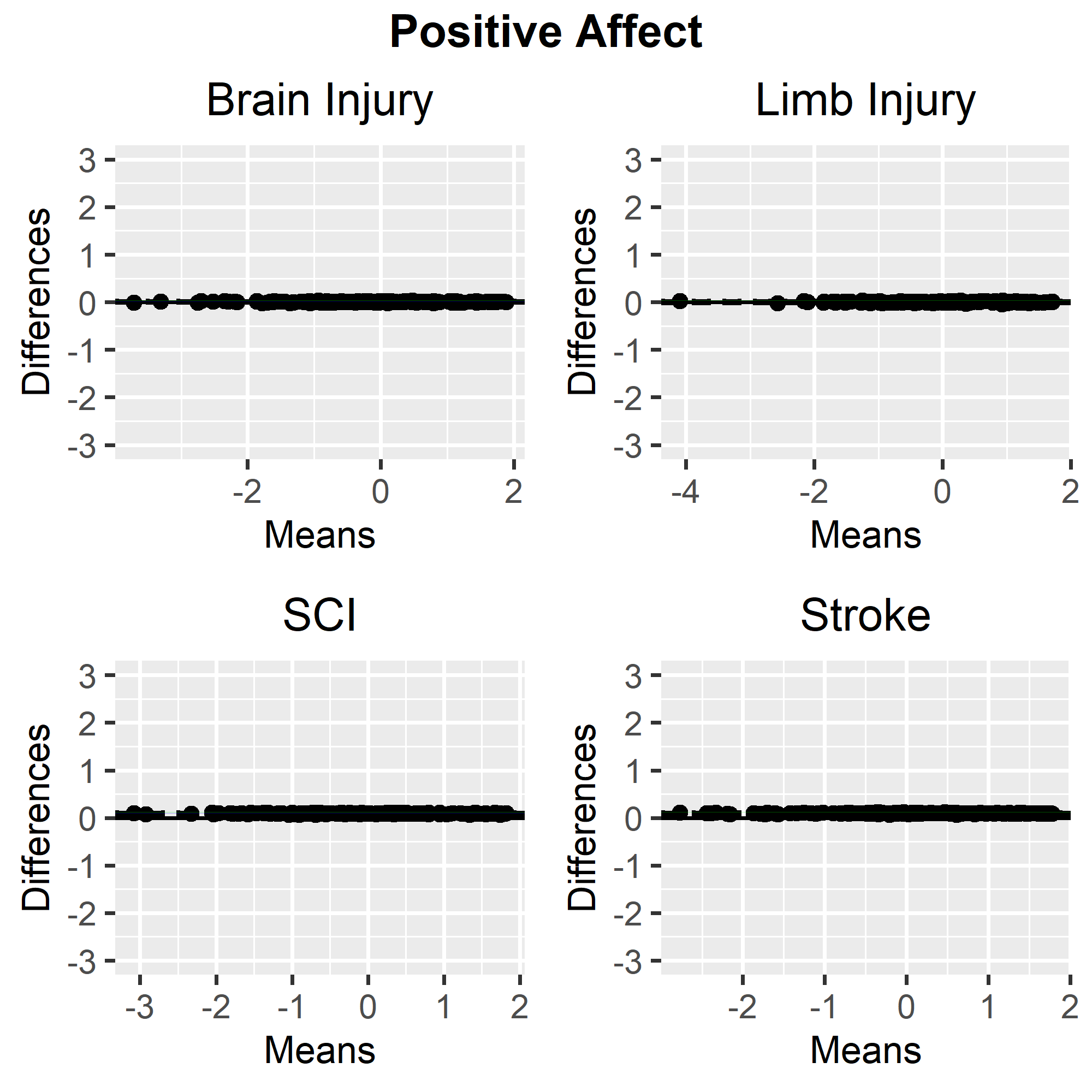


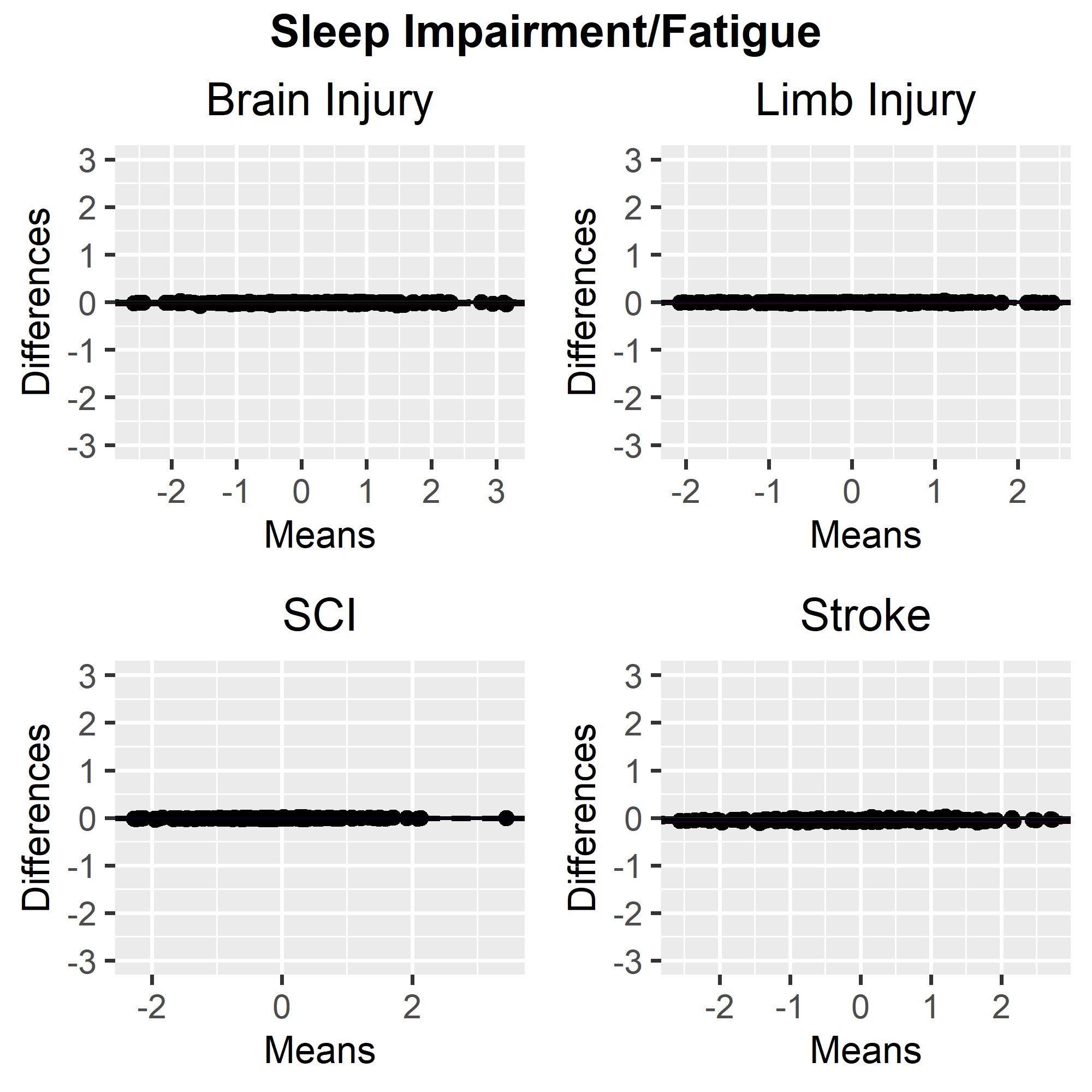


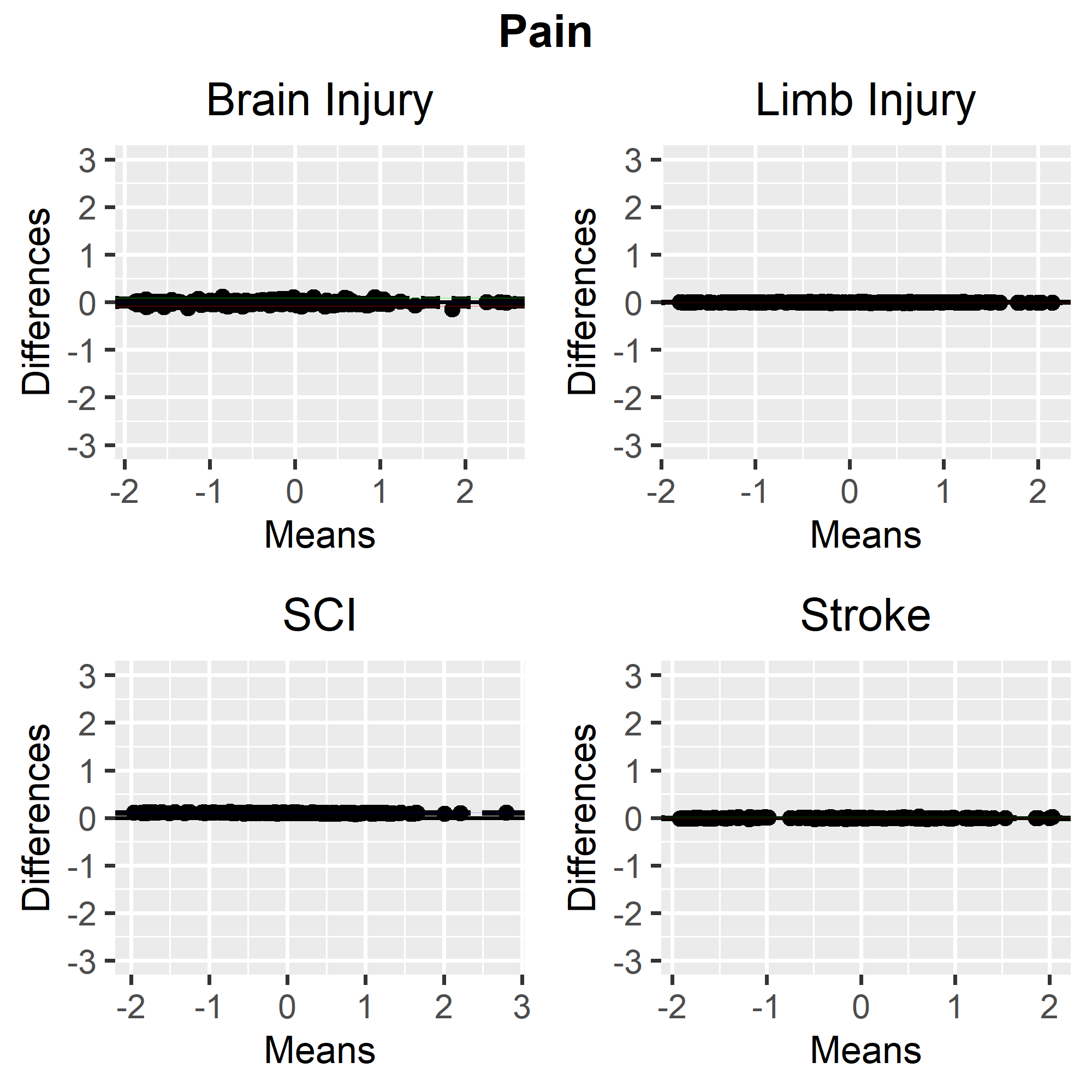


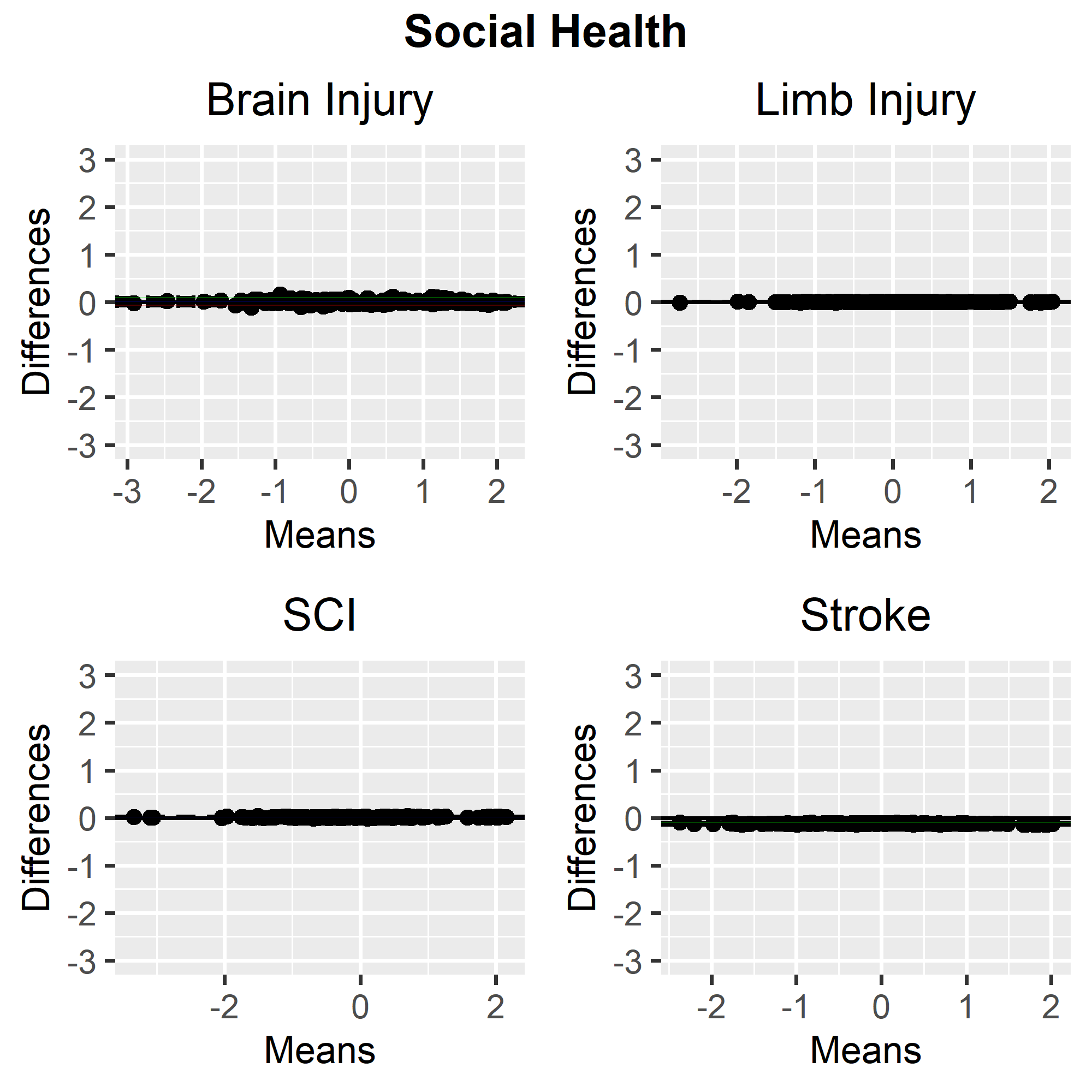


Figure S1. *Bland-Altman Plots for Comparing Full vs. Partial Scalar Invariant Models.* Each Bland-Altman plot shows differences between factors scores generated under a full scalar invariant model (with all factor loadings and intercepts constrained to equality across injury groups) and the partial scalar invariant model. The blue lines represent *bias* (average of all score differences) whereas the green and red lines represent the *limits of agreement* (range in which 95% of all score differences fall). The plots shown here were not reported in the main article because they exhibit negligible score differences between the full and partial scalar invariant models.
